# Book-Reviews

**Published:** 1860-10

**Authors:** 


### REVIEW.

*Gutta Percha as a Permanent Stopping.*—Read before the O. S., London. By HENRY LONG JACOB, M. R. C. S.

WE are in receipt of the above pamphlet, and accompanying it a small portion of the preparation. We regret that we cannot recognize any preparation as of a permanent character, in which gutta percha is the preservative agent. This preparation is remarkably white as well as remarkably light, showing a too great absence of the earthy portion, which is really the indestructible matter. Abundant experience establishes beyond question, the temporary character of any preparation of gutta percha for the mouth. We fear Mr. Jacob has not had the advantage of Dr. Hill's preparations of these materials as early as the profession in this country.

In 1852, Dr. Hill introduced his stopping five years previous to the reading of this paper, nor can we see any material difference in the two methods, except that we think the London process the longest and most tedious of the two. We are always pleased to acknowledge the advantages we have derived from various useful inventions, and are bound to acknowledge the credit in this case as entirely due to Dr. A. Hill, of Norwalk, Conn. B.

*The Anatomy of the Fifth Pair of Nerves and the Microscopical Anatomy of the Teeth.* Published by JONES & WHITE, Philadelphia.

This is a well executed colored lithograph, principally descriptive of the fifth pair of nerves, and the microscopical structure of the teeth, but also representing some of the muscles and arteries, with a correct delineation of the surrounding parts.

It is perhaps quite as creditably executed, and as full as could be reasonably expected under the circumstances. The ultimate nerve fibre is always tubular, and should have been so represented in so fine a plate. Still the mere anatomy, as here illustrated, will aid the student and refresh the memory of the more advanced. We are, however, no advocates for studying this intricate subject through the medium of plates, as such are but doubtful and rude teachers. The description accompanying this plate is the briefest possible anatomical sketch, less full, and we may say, less desirable than Cruvelhiers, Sharpe and Quains or Wilson's. All the physiology of the nerve in its office in the animal economy as well as its pathology, are omitted; indeed, it is a bare anatomical description with the least possible reference to the vital relation of the nerve. This we think in so creditable a work is a fault, for the anatomy of any portion of the nervous system is less important than its relation to the normal phenomena of life, as also to pathological action. B.

---

*The Principles and Practice of Modern Surgery.* By Robert Druitt. Philadelphia: Blanchard & Lea, 1860, 8vo, pp. 695.

Druitt's Surgery has long been a favorite with students. The facts were so well arranged and condensed into so small compass, that while the descriptions of disease and operations lost nothing in the way of clearness, they were sufficiently brief to be soon studied and easily remembered. Probably there never was a text-book more completely free from redundant verbiage. This gave it an immediate and lasting popularity, until we venture to say it is more extensively used as a text-book than any other compendium of surgical art.

The present edition is revised from the eighth London edition, published about a year ago. We are happy to see that the author has kept his book up to the time. The recent operation of excision of the knee-joint is treated as fully as the plan of the work will admit, and the opthalmoscope is described with sufficient clearness to enable the student or the country practitioner to familiarize himself with the use of the instrument, after a very little practice. A very sensible chapter on anæsthetics is appended, in which, however, Morton is made a little too prominent, to the disadvantage of Drs. Wells and Jackson. The American editor, whose modesty conceals his name, has appended a very brief paragraph in reference to hypnotism.

The American publishers bring out the work in their usual creditable style. The wood cuts are numerous and very clear and neat. They are admirably selected for the purposes of illustration, and furnish an excellent exposition of the text.

